# Loss of ATRX Protein Expression in an Aggressive Null Cell Pituitary Tumor

**DOI:** 10.1210/jcemcr/luae143

**Published:** 2024-08-06

**Authors:** Elisa Lamback, Renan Lyra Miranda, Nina Ventura, Leila Chimelli, Mônica R Gadelha

**Affiliations:** Neuropathology and Molecular Genetics Laboratory, Instituto Estadual do Cérebro Paulo Niemeyer, Rio de Janeiro 20231-092, Brazil; Neuroendocrinology Division, Instituto Estadual do Cérebro Paulo Niemeyer, Rio de Janeiro 20231-092, Brazil; Neuroendocrinology Research Center, Endocrinology Division, Medical School and Hospital Universitário Clementino Fraga Filho, Universidade Federal do Rio de Janeiro, Rio de Janeiro 21941-901, Brazil; Neuropathology and Molecular Genetics Laboratory, Instituto Estadual do Cérebro Paulo Niemeyer, Rio de Janeiro 20231-092, Brazil; Radiology Division, Instituto Estadual do Cérebro Paulo Niemeyer, Rio de Janeiro 20231-092, Brazil; Neuropathology and Molecular Genetics Laboratory, Instituto Estadual do Cérebro Paulo Niemeyer, Rio de Janeiro 20231-092, Brazil; Neuropathology and Molecular Genetics Laboratory, Instituto Estadual do Cérebro Paulo Niemeyer, Rio de Janeiro 20231-092, Brazil; Neuroendocrinology Division, Instituto Estadual do Cérebro Paulo Niemeyer, Rio de Janeiro 20231-092, Brazil; Neuroendocrinology Research Center, Endocrinology Division, Medical School and Hospital Universitário Clementino Fraga Filho, Universidade Federal do Rio de Janeiro, Rio de Janeiro 21941-901, Brazil

**Keywords:** aggressive pituitary tumor, null cell, ATRX

## Abstract

Somatic *alpha thalassemia/mental retardation syndrome X-linked (ATRX)* pathogenic variants have been shown to predict a malignant phenotype in neuroendocrine tumors. They were recently identified in aggressive pituitary tumors and carcinomas, mainly of corticotrophic origin. To our knowledge, these tumors are rare in a general cohort of pituitary tumors, with no cases described in null cell tumors. These variants can lead to loss of protein expression as revealed by immunohistochemistry. We describe a case of an aggressive null cell pituitary tumor with loss of ATRX expression. The patient underwent two transsphenoidal surgeries and radiotherapy and exhibited tumor growth despite conventional therapy. Analysis of the tumor samples revealed loss of ATRX expression in both surgical specimens, suggesting that ATRX may be a useful biomarker for the early identification of aggressive pituitary tumors.

## Introduction

Somatic *alpha thalassemia/mental retardation syndrome X-linked* (*ATRX*) pathogenic variants have been shown to predict malignant phenotypes in neuroendocrine tumors, such as pancreatic neuroendocrine tumors and pheochromocytoma/paraganglioma (1). These pathogenic variants lead to inactivation of *ATRX*, which destabilizes telomeres and facilitates the alternative lengthening of telomeres, resulting in cell immortality (1). The presence of these *ATRX* pathogenic variants can be assessed by genetic sequencing or loss of protein immunostaining. In pituitary tumors, these pathogenic variants are unusual in an unselected cohort, as shown by a study that assessed 248 patients with different types of pituitary tumors and carcinomas (2). However, they have been detected in 19% of aggressive tumors and carcinomas, mainly of the corticotroph lineage (1). In nonfunctioning pituitary tumors, *ATRX* pathogenic variants are extremely rare (1). We describe the case of a patient who had an aggressive null cell pituitary tumor with loss of ATRX protein expression.

## Case Presentation

A male patient presented at the age of 63 with holocranial headache that had progressed for five months and was associated with progressive vision loss. On a confrontational visual field, he exhibited right inferior temporal quadrantanopia. He did not have features of acromegaly or signs of Cushing syndrome.

## Diagnostic Assessment

Biochemical findings revealed panhypopituitarism: total testosterone 2.6 nmol/L (75 ng/dL [reference range, 832.1-2877.7 nmol/L; 240-830 ng/dL]), free thyroxine 9.0 pmol/L (0.70 ng/dL [reference range, 10.3-24.5 pmol/L; 0.80-1.90 ng/dL]), insulin-like growth factor I (IGF-I) 8.2 nmol/L (63 ng/mL [reference range, 2.6-26.7 nmol/L; 20-204 ng/mL]), morning serum cortisol 198.6 nmol/L (7.2 mcg/dL [reference range, 83-414 nmol/L; 3-15 mcg/dL]), and non-tumoral hyperprolactinemia as evidenced by diluted prolactin concentration of 21.3 mcg/L (reference range, 2.0-15.2 mcg/L).

Sellar magnetic resonance imaging (MRI) revealed an expansive sellar lesion with suprasellar and right parasellar extensions of 3.3 × 3.6 × 2.5 cm abutting the optic chiasm (Knosp 3 on the right side). He was diagnosed with a nonfunctioning pituitary tumor (NFPT).

## Treatment

He underwent transsphenoidal surgery. Hormone replacement with levothyroxine 100 mcg/day, prednisone 2.5 mg/day, and intramuscular testosterone cypionate 200 mg every 21 days was administered. An insulin tolerance test confirmed adrenal insufficiency. During follow-up, the residual mass grew (> 20% tumor volume), his vision deteriorated, and right ptosis developed. He underwent another transsphenoidal surgery at the age of 65. Sellar MRI revealed a residual tumor in the right cavernous sinus, and the patient underwent 28 sessions of stereotactic radiotherapy (5040 Gy).

## Outcome and Follow-Up

**Figure 1.**
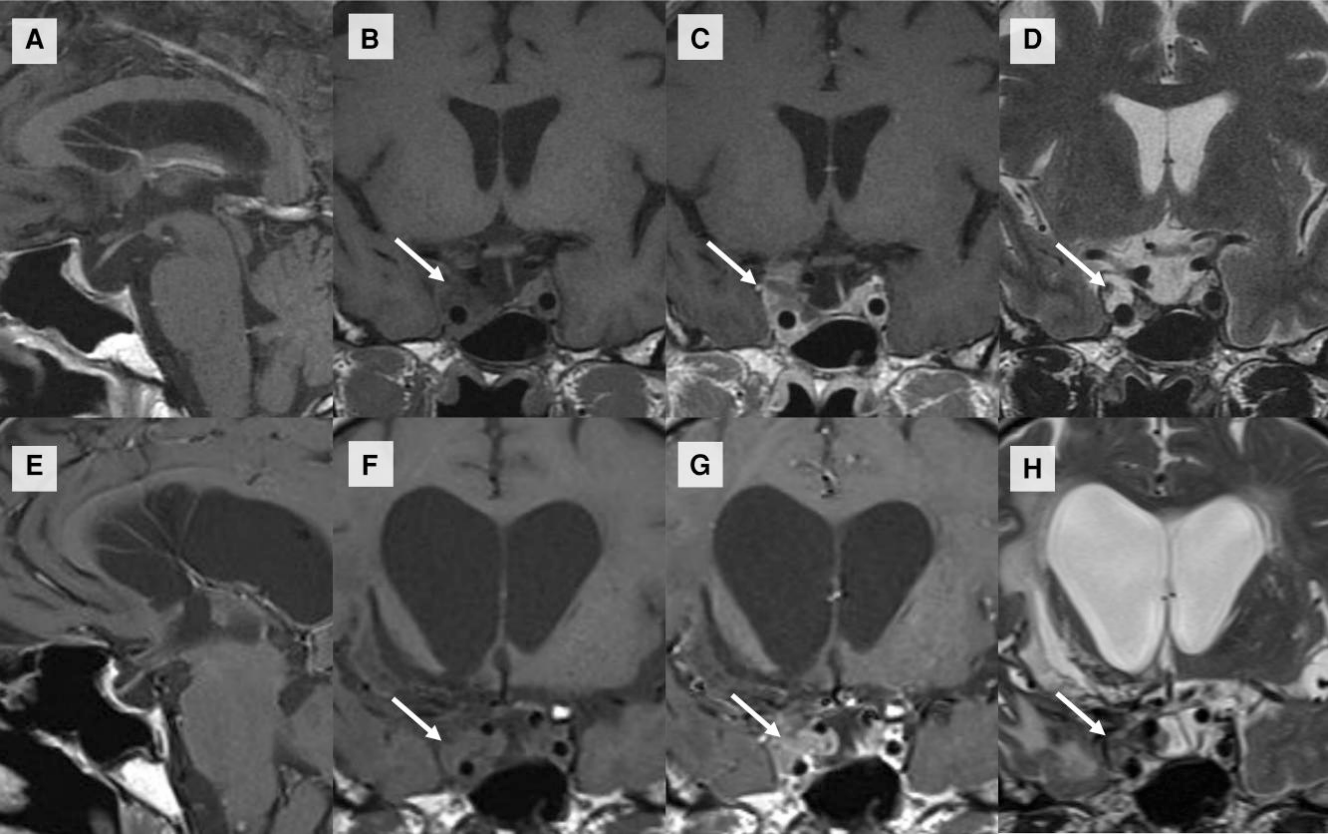
Magnetic resonance (MR) imaging of the sella turcica. Sagittal T1 (A), coronal T1 (B), coronal T1 postcontrast (C), and coronal T2 (D) images showing a residual tumor in the right cavernous sinus (arrows), predominantly isointense in T1 and hyperintense in T2, with contrast enhancement. The normal pituitary gland and pituitary stalk were displaced to the left side. Sagittal T1 (E), coronal T1 (F), coronal T1 postcontrast (G) and coronal T2 (H) images on the last MR image revealing growth of the residual tumor after radiotherapy (arrows) and a dilated third ventricle.

Four years after radiotherapy, the residual mass on the right cavernous sinus grew from 1.3 × 1.2 × 1.6 cm (volume 1.3 cm^3^) to 2.0 × 1.9 × 1.6 cm (volume 3.2 cm^3^) (Fig. 1), and we opted for conservative management because he developed multiple sequelae: intracranial hypertension, ischemic stroke with hemorrhagic transformation requiring trepanation, and external ventricular drainage. Computed tomography of the abdomen and thoracic radiography were normal, indicating that no metastasis was visible, although other, more specific, imaging modalities were not used.

At his last appointment, at 70 years old, five years after radiotherapy, he was fragile and debilitated by multiple treatments and a prolonged hospital stay. He was lost to follow-up and has been unreachable for the last two years.

**Figure 2.**
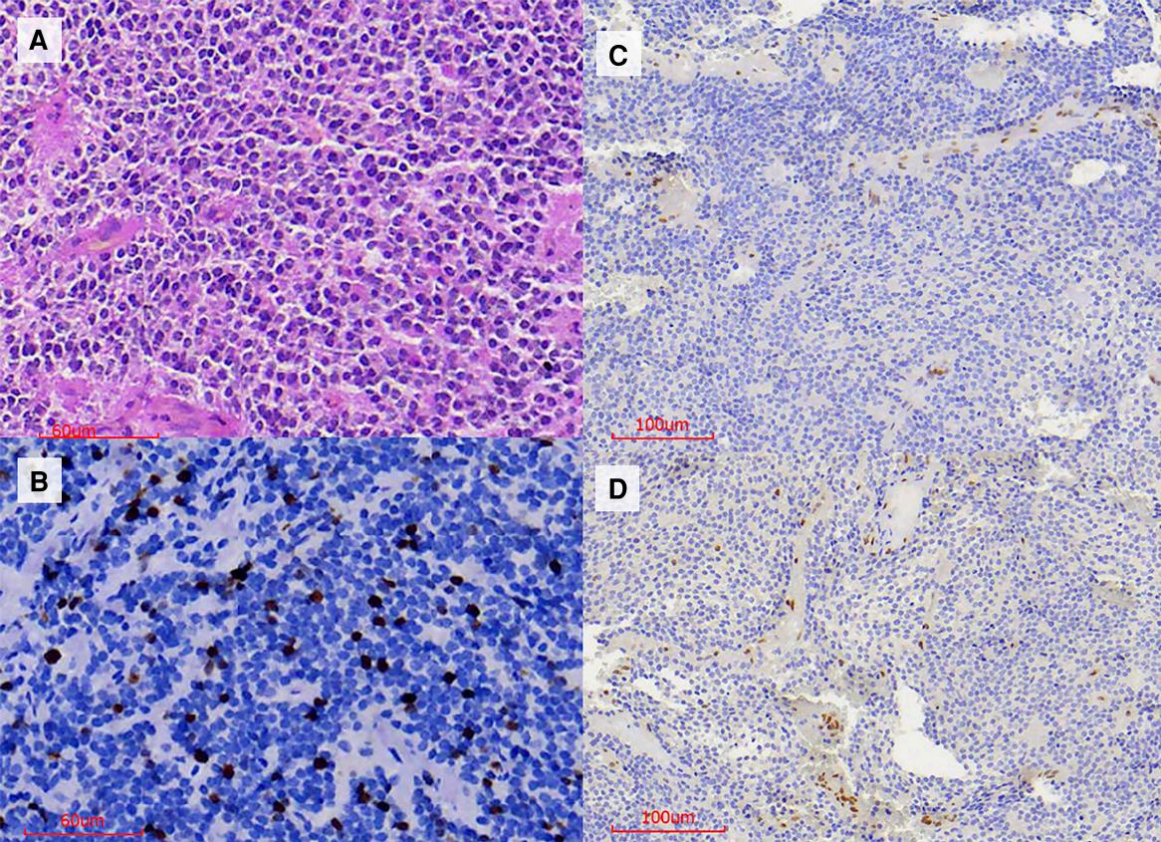
Histopathological analysis. A, Hematoxylin and eosin staining showing neoplasia characterized by diffuse proliferation of uniform cells with chromophobe cytoplasm. B, Ki-67 of 11% in “hotspots.” C, Negative ATRX immunostaining in neoplastic cells from the second surgery. Positive endothelial cells served as an internal control. D, Negative ATRX immunostaining in neoplastic cells from the first surgery. Positive endothelial cells served as an internal control.

As previously published, pathological examination included histopathological and immunohistochemical studies (3). Histopathological examination revealed a pituitary tumor of null cell origin (negative hormone, alpha-subunit and transcription factors: steroidogenic factor [SF-1], T-box family member TBX19 [T-PIT], and pituitary-transcription factor 1 [PIT-1]), synaptophysin (+), a Ki-67 index of 11%, and 7 mitoses/10 high-power fields (Fig. 2A-2B). ATRX protein immunoexpression in the second surgical specimen was negative (Fig. 2C). Since ATRX expression was lost in the last surgery, we investigated its expression pattern in the first surgery and found that it was also negative in the surgical sample from the first surgery (Fig. 2D).

## Discussion

Loss of ATRX protein expression has been recently described in aggressive pituitary tumors and carcinomas, mainly of corticotrophic and lactotrophic origins (1, 2, 4-7) (Table 1). They appear to be extremely rare in nonfunctioning pituitary tumors and have not been reported, to the best of our knowledge, in null cell tumors. We described the case of a patient harboring an aggressive null cell tumor that was resistant to conventional therapy, with loss of ATRX protein expression in both surgical specimens.

**Table 1.** Literature review of studies analyzing ATRX (gene and/or protein expression) in pituitary tumors

Author, year	Patients’ characteristics	ATRX protein expression loss	*ATRX* mutation status
Casar-Borota, 2017 (2)	General, unselected cohort	Total: 1/248 Metastatic functioning ACTH carcinoma: 1/2*^a^* NFPT: 0/152 (FSH/LH: 117, null cell: 15, ACTH: 15, PRL: 2, alpha-subunit: 3) GH: 0/60 ACTH: 0/18 Nelson syndrome: 0/4 PRL: 0/9 TSH: 0/2 Plurihormonal GH/PRL/TSH: 0/1	*ATRX* mutation in the pituitary specimen of the ATRX-immunonegative tumor
Guo, 2018 (7)	55-year-old male with a corticotroph carcinoma with lung metastases	NA	*ATRX* mutation in the pituitary specimen
Chen, 2019 (5)	Children and adolescents with pituitary tumors	Total: 3/42 PRL: 3/18 ACTH: 0/9 GH: 0/6 NFPT: 0/9	NA
Heaphy, 2020 (4)	General, unselected cohort	Total: 1/106 NFPT: 1/82*^b^* GH: 0/15 PRL: 0/3 GH-PRL: 0/2 Corticotroph: 0/2 Unknown: 0/2	No *ATRX* mutation
Casar-Borota, 2021 (1)	Aggressive pituitary tumor or pituitary carcinoma	Total: 9/48 (19%) with 8 total loss and 1 partial loss of ATRX ACTH: 7/22 (32%), of which 6 were functioning*^a^* and 1 silent PRL: 1/15 GH-PRL: 1/2 GH: 0/4 Silent PIT-1: 0/3 Plurihormonal TSH/FSH: 0/1 Null cell: 0/1	*ATRX* mutations in all 9 ATRX-immunonegative tumors
Sumislawski, 2022 (6)	53-year-old male with a corticotroph carcinoma with liver and vertebral metastases	ATRX protein expression retained in the pituitary and liver specimens	*ATRX* mutation in the liver metastases, not in the pituitary tumor
Present study	63-year-old male patient with an aggressive null cell tumor	Loss in both surgical specimens	NA

Abbreviations: ACT, adrenocorticotrophic hormone; ATRX, alpha thalassemia/mental retardation syndrome X-linked; FSH, follicle-stimulating hormone; GH, growth hormone; LH, luteinizing hormone; NA, not available; NFPT, nonfunctioning pituitary tumor; PRL, prolactin; TSH, thyroid-stimulating hormone.

^*a*^Same patient.

^*b*^Negative hormones. No transcription factors were evaluated.

Loss of ATRX protein expression was first identified in a pituitary tumor in 2017 (2). In this study, 248 patients were selected, and the loss of ATRX was observed in 1 of 2 patients with metastatic functioning corticotroph carcinoma (Table 1). In the other carcinoma patient, and in the general cohort composed of different types of pituitary tumors, including 152 nonfunctioning pituitary tumors, no protein loss was observed (Table 1). An inactivating *ATRX* variant was identified in the pituitary specimen of the ATRX-immunonegative tumor.

Another study that analyzed ATRX expression in an unselected cohort of pituitary tumors revealed loss of protein expression in only 1 of 106 cases analyzed (4). This patient was a 73-year-old male with a recurrent nonfunctioning hormone-negative pituitary tumor of unknown origin. Since transcription factors were not analyzed and 95% of hormone-negative pituitary tumors have a pituitary lineage as determined by transcription factor assessment, these nonfunctioning pituitary tumors were likely not null cell tumors (8). In this case, next-generation sequencing was performed, but no mutation in the *ATRX* gene was detected (4). Therefore, in a general cohort of pituitary tumors, the loss of ATRX protein expression is rare but seems to be related to a worse prognosis.

The first group that analyzed ATRX expression in unselected pituitary tumors investigated whether loss of ATRX protein expression was present in a cohort with aggressive pituitary tumors and carcinomas (1). In this study, the authors included the carcinoma of the corticotroph lineage that was previously published (2). They found that 19% (9/48) of samples had loss of ATRX protein expression, which was confirmed by next-generation sequencing. The patients had a median age at diagnosis of 45 years (ranging from 23 to 72) and were predominantly male (67%). Seven cases were of corticotrophic origin, one case was of lactotrophic origin, and the other case was of somatolactotroph origin. One null cell pituitary tumor (with negative hormone and transcription factor expression) that displayed intact ATRX expression was analyzed.

Another case report described the loss of ATRX expression in corticotroph carcinoma tissue from a 55-year-old male patient (7). In another 53-year-old male patient with a corticotroph tumor, an *ATRX* variant was detected in the liver metastasis but not in the primary tumor (6). In this case, the ATRX protein was not altered by the *ATRX* variant. In the pediatric population, loss of ATRX expression has been identified in 7% (3/42) of patients. These cases were of lactotrophic origin, with no relationship to prognosis (with a follow-up period of 2 to 24 months) (5).

Therefore, loss of ATRX protein expression can occur in patients from a wide age range but seems to be more prevalent in tumors of corticotrophic lineage, which exhibit more aggressive behavior (9). Regarding null cell tumors, defined by the World Health Organization classification (10) as tumors that are negative for hormones and transcription factors, we identified, for the first time, loss of ATRX protein expression in an aggressive null cell tumor (10, 11). The ATRX loss was present since the first surgery, suggesting that *ATRX* may act as a tumorigenic driver and allow for the early identification of aggressive tumors.

Interestingly, this patient's tumoral sample was analyzed in another study from our group, in which it was revealed that it had increased cyclin A mRNA expression (3). In this study, cyclin A expression was studied in nonfunctioning pituitary tumors and was found to be lower in the vast majority of tumoral samples than in normal pituitary samples. This patient's tumor had the highest mRNA expression (fold change of 1.9) of the entire cohort. These findings suggest that the tumoral cells were proliferating, as indicated by the increased expression of cyclin A, a cell cycle marker responsible for DNA replication and synthesis, and the initiation of mitosis (3). Additionally, the tumor had a high proliferation index (Ki-67 above 10%), which reinforces this hypothesis. Furthermore, radiotherapy was not able to slow cell division since the residual mass grew, possibly because of the loss of ATRX.

In conclusion, this case describes the loss of ATRX expression in an aggressive null cell pituitary tumor, for the first time. Loss of expression was present after the first surgery, suggesting that ATRX could be used as a marker for early identification of aggressive behavior in pituitary tumors.

## Learning Points

- Loss of ATRX protein expression is present in aggressive pituitary tumors and carcinomas.
- Loss of ATRX protein expression was identified for the first time in an aggressive null cell pituitary tumor.
- ATRX immunostaining may be a useful biomarker for the early identification of aggressive pituitary tumors.

## Data Availability

Original data generated and analyzed during this study are included in this published article.

## Abbreviations

ATRX: alpha thalassemia/mental retardation syndrome X-linked
MRI: magnetic resonance imaging
